# OLDER US ADULTS PREFER SHORT, FREQUENT RESISTANCE TRAINING PROGRAMS, ESPECIALLY THOSE WITH DIFFICULTY WALKING

**DOI:** 10.1093/geroni/igad104.3506

**Published:** 2023-12-21

**Authors:** Jordan Kurth, Christopher Sciamanna, Cheyenne Herrell, Margaret Danilovich, David Conroy, Kathryn Schmitz, Matthew Silvis, Matthew Ladwig

**Affiliations:** The Pennsylvania State University College of Medicine, Hershey, Pennsylvania, United States; The Pennsylvania State University College of Medicine, Hershey, Pennsylvania, United States; The Pennsylvania State University College of Medicine, Hershey, Pennsylvania, United States; CJE SeniorLife, Chicago, Illinois, United States; Penn State University, University Park, Pennsylvania, United States; University of Pittsburgh, Pittsburgh, Pennsylvania, United States; The Pennsylvania State University College of Medicine, Hershey, Pennsylvania, United States; Purdue University Northwest, Hammond, Indiana, United States

## Abstract

Though resistance training (RT) improves strength and physical function, fewer than 20% of older adults do RT at least twice weekly, as recommended by national guidelines. Even when programs are free, such as Silver Sneakers, fewer than 25% participate and those that do rarely attend (averaging < 20 visits/year). Many individuals cite “lack of time” as the primary reason for not attending. Therefore, one approach to increase participation in RT programs could be making sessions shorter. However, little is known about older adult RT program preferences. Therefore, we conducted a nationwide survey among 611 US adults aged 65 and over. We compared their interest in a traditional (45 minutes, three days/week) RT program to a shorter, more frequent (5-minute, every day) RT program. Overall, 2.2 times as many older adults preferred the shorter vs. the traditional RT program (68.4% v. 31.6%; p< 0.001). Furthermore, barrier self-efficacy for the shorter program was 50% greater than the traditional program. Adults with health concerns, like poorer self-rated health and a history of falls, were significantly more likely to be interested in the shorter program. Notably, 87% of older adults with difficulty walking also preferred the shorter program. These findings suggest strong interest in short RT programs among older adults, especially those with poorer health and mobility disabilities for whom traditional RT programs may be too challenging. Reducing the duration of RT programs may be an appealing option to engage older adults in RT, particularly among those for whom the program would provide the most benefit.

